# Multifunctional Nanofibrous Dressing with Antimicrobial and Anti-Inflammatory Properties Prepared by Needle-Free Electrospinning

**DOI:** 10.3390/pharmaceutics13091527

**Published:** 2021-09-21

**Authors:** Laura Victoria Schulte-Werning, Anjanah Murugaiah, Bhupender Singh, Mona Johannessen, Rolf Einar Engstad, Nataša Škalko-Basnet, Ann Mari Holsæter

**Affiliations:** 1 Drug Transport and Delivery Research Group, Department of Pharmacy, Faculty of Health Sciences, UiT The Arctic University of Norway, 9037 Tromsø, Norway; laura.v.schulte-werning@uit.no (L.V.S.-W.); m.anju11@live.no (A.M.); natasa.skalko-basnet@uit.no (N.Š.-B.); 2 Research Group for Host-Microbe Interaction, Department of Medical Biology, Faculty of Health Sciences, UiT The Arctic University of Norway, 9037 Tromsø, Norway; bhupender.singh@uit.no (B.S.); mona.johannessen@uit.no (M.J.); 3 Biotec BetaGlucans AS, 9019 Tromsø, Norway; rolf.engstad@biotec.no

**Keywords:** nanofiber, Nanospider^TM^, electrospinning, chloramphenicol, antimicrobial activity, anti-inflammatory activity

## Abstract

An active wound dressing should address the main goals in wound treatment, which are improved wound healing and reduced infection rates. We developed novel multifunctional nanofibrous wound dressings with three active ingredients: chloramphenicol (CAM), beta-glucan (βG) and chitosan (CHI), of which βG and CHI are active nanofiber-forming biopolymers isolated from the cell walls of *Saccharomyces cerevisiae* and from shrimp shells, respectively. To evaluate the effect of each active ingredient on the nanofibers’ morphological features and bioactivity, nanofibers with both βG and CHI, only βG, only CHI and only copolymers, polyethylene oxide (PEO) and hydroxypropylmethylcellulose (HPMC) were fabricated. All four nanofiber formulations were also prepared with 1% CAM. The needle-free Nanospider^TM^ technique allowed for the successful production of defect-free nanofibers containing all three active ingredients. The CAM-containing nanofibers had a burst CAM-release and a high absorption capacity. Nanofibers with all active ingredients (βG, CHI and CAM) showed a concentration-dependent anti-inflammatory activity, while maintaining the antimicrobial activity of CAM. The promising anti-inflammatory properties, together with the high absorption capacity and antimicrobial effect, make these multifunctional nanofibers promising as dressings in local treatment of infected and exuding wounds, such as burn wounds.

## 1. Introduction

Infections are one of the main local causes of impaired wound healing, leading to increased morbidity [1]. Wound infections, e.g., infections in burn wounds, are often treated effectively by applying antibiotics directly onto the wound bed [2]. The local administration of antibiotics is favourable, since it allows effective and selective treatment, lowers the systemic drug exposure and thus reduces the risk of systemic side effects [3,4]. Local drug treatment is also preferable with respect to antibiotic resistance since antibiotics restricted to topical use, e.g., chloramphenicol (CAM), seem to have a lower resistance rate and to maintain their activity against several otherwise resistant organisms [5]. CAM is an old, broad-spectrum antibiotic that inhibits bacterial growth by binding to the 50S ribosomal subunit. Despite its broad-spectrum antimicrobial action, its use has been limited due to its side effects (e.g., bone marrow toxicity), which has discouraged its use in systemic treatment [6]. Today, CAM is mainly used in the topical treatment of eye infections [7], but also systemically in the treatment of severe infections (e.g., bacterial meningitis) [8].

For an optimal topical antibiotic therapy, an efficient delivery system with the embedded antimicrobial agent(s) is crucial [9]. In this study, we explored nanofibrous dressings formed by electrospinning for this purpose, since nanofibers previously have shown promising features in the local delivery of antimicrobial agents [10]. Nanofibrous wound dressings are polymer mats comprising a net of polymer fibers with diameters within the nanometre range [11]. Nanofibers are potentially beneficial as wound dressings for at least three reasons. Firstly, they have a high surface-area-to-volume ratio and porosity, which facilitate oxygen and water permeation through the fibers and allow high absorption of wound fluid [12]. Secondly, the fibrous structure resembles the extracellular matrix and can thus facilitate cell ingrowth and proliferation [13]. Thirdly, the nanofiber formation process allows the use of bioactive polymers as matrix and enables the incorporation of additional active ingredients, e.g., antibiotics, anti-cancer agents and antioxidants, resulting in multifunctional dressings with advantageous properties in local drug delivery [11,14,15].

The most convenient way to produce nanofibers is by needle-electrospinning. This simple and flexible technique produces nanofibers from different polymers and offers several possibilities to tune the morphology and function of the spun nanofibers [16]. However, the slow fiber production rate of the needle-electrospinning machine limits the utility of this technique in an industrial scale. Alternative and more efficient electrospinning techniques have thus been developed [17,18,19,20]. Among them are the “needle-free electrospinning techniques”, such as the Nanospider^TM^ technique with a stationary electrode wire used in this study. This technology offers several benefits (i) a continuous fiber production, (ii) it is faster than the traditional needle-electrospinning technique, and (iii) it might allow industrial-scale production of nanofibers [21,22,23,24,25]. Furthermore, this technology avoids typical problems of needle-electrospinning (e.g., needle clogging) [26] and the machine is easier to clean [22]. Another potential advantage of wire electrospinning is the high charge density on the surface of the electrospinning wire that can facilitate the formation of thinner nanofibers [27], which might be beneficial since a fiber size below the bacterial size has been shown to limit bacterial adhesion and spreading for *P. aeruginosa* and *E. coli* [28]. However, the electrospinning process in needle-free electrospinning is not aided by capillary forces, which occur in needle-electrospinning, and is therefore more difficult to control [26]. A common problem of needle-free electrospinning systems is to achieve a uniform electric field along the spinneret, which can result in non-uniform fibers. However, among the different needle-free electrospinning systems, the wire and cylinder systems have a high uniformity of the electric field. Thus, fibers with an even diameter distribution are normally obtained with these setups [27].

Both natural and synthetic polymers can be used in electrospinning [11]. Natural polymers are often biodegradable and biocompatible and many are known to have antimicrobial, immunostimulating, or anti-inflammatory effects [11]. Furthermore, wound dressings made from natural polymers can mimic biological systems and form dressings with reduced risk of immunological reactions [29]. In this current study, two natural polymers with beneficial properties in wound healing were selected for nanofiber formation. Beta-glucans (βG) are glucose polymers that can be derived from different sources such as bacteria, grain, mushrooms, and yeast [30]. The poor water solubility of βG demands further processing to obtain water-soluble βG. This study used water-soluble β-1.3/1.6 glucan (SBG^®^). SBG^®^ contains 2.5% βG (*w*/*w*) in water and forms a gel at room temperature [31,32]. The βG in SBG^®^ is originally extracted from the cell walls of *Saccharomyces cerevisiae*, and is thereafter purified and hydrolysed by a patented method to obtain water-soluble βG with a mean MW of 7 × 10^5^. SBG^®^ has previously been proven beneficial as an active ingredient in the topical treatment of diabetic foot and leg ulcers [33]. Grip et al. fabricated SBG^®^-nanofibers successfully with the same technology as the one applied in this study [23]. We aimed to obtain multifunctional nanofibers with not only the immunostimulating effect gained from SBG^®^ but also with antimicrobial potential, making the nanofibrous wound dressing suitable for the treatment of infected wounds. The antimicrobial activity was targeted based on the known antibiotic CAM, as well as a second bioactive polymer, chitosan (CHI), exhibiting intrinsic antimicrobial activity. In this way, the SBG^®^-nanofibers, proven to have high wound healing potential in diabetic mice [23], in addition it should be able to act on infection as well as biofilm formation. CHI is a natural polymer derived from chitin by deacetylation. CHI is non-toxic, biocompatible, and biodegradable and exhibits antimicrobial properties, all of which make it beneficial in wound healing [34]. CHI-hydrogels have previously been shown to have antimicrobial activity against bacteria found in infected wounds, e.g., *S. aureus* [35,36]. CHI has been electrospun into nanofibers earlier, as wound dressing material or as a drug delivery system. Deng et al. reported antimicrobial activity of CHI/PEO nanofibers with lauric arginate [37], while Barzegar et al. fabricated core-shell nanofibers loaded with essential oils with CHI/polyvinyl alcohol as core and poyvinylpyrrolidone/maltrodextrin as shell that also showed antimicrobial activity [38].

Although both CHI and βG have been individually electrospun into nanofibers before, the combination of both polymers in one nanofibrous dressing is novel. To our knowledge, this is also the first study investigating the incorporation of CAM via electrospinning into nanofibers containing either CHI or βG. Furthermore, the number of studies published on needle-free electrospinning is still limited [26,39]. More knowledge on the electrospinning of these polymers when utilizing these spinning methods is needed to meet their future potential.

Since the spinnability of natural polymers is often poor, especially for CHI, co-polymers are often applied in electrospinning to improve the spinnability and mechanical properties of the fibers [11]. We chose the same co-polymers as the ones applied by Grip et al. [23]: Polyethylene oxide (PEO) and hydroxypropylmethylcellulose (HPMC). PEO is a synthetic biocompatible and biodegradable polymer that is known to enhance the spinnability of CHI [40]. HPMC is a cellulose-based polymer, often used for mucosal and buccal applications and has a high swelling capacity. These characteristics make HPMC an ideal polymer for use in highly absorbing wound dressings [41]. The choice of solvent is important for the spinning process, and toxic solvents (e.g., trifluoro acetic acid, dichloromethane) must be avoided, especially in an open system such as the Nanospider^TM^. Toxic solvents are not only bad for the safety of the operator and the environment but might also affect the safety of the nanofibers negatively if solvent residues are not removed from the fibers [42].

This study used a novel combination of active ingredients, combining CHI, known for its antimicrobial and wound healing properties, with the immunostimulating βG and the antibiotic CAM. Hereby, we aimed at developing a multifunctional nanofibrous wound dressing with a novel combination of features with known relevance for wound healing, exhibiting both antimicrobial and anti-inflammatory activity. To judge its appropriateness in wound healing, we determined features relevant to wound dressing, such as mechanical strength, swelling index and CAM release. Finally, we also tested cell toxicity and antimicrobial and anti-inflammatory activity in vitro.

## 2. Materials and Methods

### 2.1. Materials

Chitosan (CHI) from shrimp (Mw 426 kDa, deacetylation degree 87.4%) and soluble beta-1,3/1,6-glucan (βG) (SBG^®^; 2.5% (*w*/*w*) aqueous hydrogel) were a generous gift from Chitinor AS (Tromsø, Norway) and Biotec BetaGlucans AS (Tromsø, Norway), respectively. Hydroxypropylmethylcellulose (HPMC) (Benecel^TM^ E4M HPMC) was obtained from Ashland (Covington, KY, USA). Polyethylene oxide (PEO), with an average molecular weight of 900,000 g/mol was manufactured by Dow Chemical Company (Midland, MI, USA). Albunorm^®^ (human serum albumin, 200 g/L) was produced by Octapharma AG (Lachen, Switzerland). Cell-counting kit-8, RPMI-1640 medium with L-glutamine and sodium bicarbonate, fetal bovine serum (FBS), penicillin-streptomycin, acetic acid (<99.9%), lipopolysaccharides (from *Escherichia coli* O55:B5), sulphanilamide, N-(1-Naphtyl)ethylenediamine dihydrochloride, ethanol (96% *v*/*v*), chloramphenicol (≥98%), sodium chloride, sodium phosphate dibasic dihydrate (Na_2_HPO_4_ · 2H_2_O) and potassium phosphate monobasic (KH_2_PO_4_) were purchased from Sigma-Aldrich (St. Louis, MO, USA). Ortho-phosphoric acid ≥85% was acquired from Kebo Lab Ab (Oslo, Norway). Dulbecco’s Modified Eagle Medium (DMEM) High Glucose was purchased from Biowest (Nuaillé, France). Chloramphenicol Antimicrobial Susceptibility discs (30 µg, Oxoid^TM^) were acquired from Thermo Fisher Scientific Inc. (Waltham, MA, USA). *Escherichia coli* ATCC 25922, *Staphylococcus aureus* ATCC 25923 and murine macrophages (RAW 264.7 cells) were obtained from ATCC (Manassas, VA, USA). Human keratinocytes (HaCaT cells) were acquired from AddexBio (San Diego, CA, USA). The substrate used for electrospinning (polypropylene blue spunbond) was delivered by Elmarco (Liberec, Czech Republic).

### 2.2. Nanofiber Preparation

#### 2.2.1. Polymer Solution Preparation

Eight different polymer solutions were prepared. The total dry content of all-polymer solutions was kept at 2.5% (*w*/*w*). All polymer solutions were prepared with the same solvents, with a final concentration of 60% (*w/w*) ethanol, 3% (*w/w*) acetic acid and 34.5% (*w*/*w*) water. The polymer composition, as well as the CAM-content of the different polymer solutions, is presented as % dry content in Table 1. All polymer solutions contained the co-polymers PEO and HPMC (ratio of 1:1) and optional βG and/or CHI as active polymers. Thus, when βG and CHI were added, the content of PEO and HPMC was reduced accordingly. The different polymers and active ingredients were dissolved and hydrated: CHI was dissolved in a mixture of the appropriate amount of SBG^®^, water and acetic acid and heated on a magnetic stirrer overnight at 50 °C. Since PEO is soluble in ethanol when heated [43], 5% (*w*/*w*) PEO was first dispersed in ethanol, and further dissolved under constant stirring at 70°C for at least 80 min. The 5% PEO solution and the βG and/or CHI-containing solution were added to an HPMC-dispersion in ethanol. The amount of HPMC and ethanol was adjusted to achieve a final ethanol concentration of 60% (*w*/*w*) and the PEO content wanted for the specific formulation (Table 1). All solutions were stirred overnight at 50 °C and allowed to rest one day at room temperature prior to electrospinning. CAM was dissolved in ethanol (10 mg/mL) and this solution was added to the polymer solution on the day of the electrospinning when a final CAM concentration of 1% was targeted (Table 1).

#### 2.2.2. Solution Characterisation

Conductivity of the polymer solutions was measured using a Sension^TM^+ EC7 Basic Conductivity laboratory Kit (Hach Company, Loveland, CO, USA). Surface tension was determined applying the Du Noüy ring method with a Pt-Ir-ring K 610 (Krüss GmbH, Hamburg, Germany) using a K6 force tensiometer (Krüss GmbH, Hamburg, Germany).

The viscosity of the solutions was measured applying the IKA^®^ Rotavisc hi-vi II Complete (IKA^®^-Werke GmbH & Co. KG, Staufen, Germany), using the spindle DIN-SP-6 with the sample holder DIN-C-2 (IKA^®^-Werke GmbH & Co. KG, Staufen, Germany). The measurement time was one minute with a rotational speed of 20 rpm. The viscosity was recorded as a function of the shear stress over the shear rate.

The pH was examined using a Fisherbrand™ accumet™ AP115 Portable pH Meter Kit (Fisher Scientific, MA, USA). All measurements were performed in triplicates.

#### 2.2.3. Electrospinning of Polymer Solutions

The polymers solutions were electrospun using the Elmarco Nanospider^TM^ NS Lab machine (Elmarco, Liberec, Czech Republic), equipped with a stationary spinning electrode wire with an applicable spinning voltage of 80 kV DC current with a unidirectional substrate unwind/rewind. The substrate speed was 2 mm/min and the wire-to-collector distance was kept at the maximum distance of 24 cm. A one-sided carriage (40 mL), filled with the polymer solution, coated the electrospinning wire at its maximum speed of 300 mm/s. All solutions were spun for 80 min in total. The carriage was refilled with polymer solutions every 20 min. The relative humidity was in general targeted to be between 24 and 29%, but one out of three replicates was spun at a higher humidity of 39 and 40% for two formulations (βG-CAM-nf and CHI-CAM-nf), respectively. The temperature was kept at 24 ± 2 °C. For analysis, the nanofibrous mat was always removed from the substrate.

### 2.3. Nanofiber Characterisation

#### 2.3.1. Morphology and Fiber Diameter

Morphology of the nanofibers was examined by Field Emission Scanning Electron Microscopy (FE-SEM) using the Zeiss Sigma FE-SEM microscope (Carl Zeiss, Oberkochen, Germany). The specimens were dried in a desiccator overnight prior to measurement. A double-sided carbon tape was applied to mount the specimens on the stubs before coating with gold/palladium using a Polacron SC7640 high-resolution sputter coater (Quorum Technologies LTD, Kent, UK). All samples were taken from the middle of the nanofibrous mat. Fiber diameter was analysed using ImageJ [44]. Three pictures were analysed for each fiber formulation and 100 fiber-diameters were measured manually on each picture.

#### 2.3.2. Tensile Properties

Tensile properties of the nanofibers were investigated applying the Texture Analyzer TA.XT plus (Stable Micro Systems Ltd., Surrey, UK). The method was based on the ASTM-Standard D882-18 [45]. The thickness of the specimens was determined using an IP54 Digital Micrometer (Wilson Wolpert Instruments, Aachen, Germany) and the measured thickness was inserted into the software prior to tensile strength measurement. Specimens in dimensions of 10 × 80 mm were placed between two tensile grips with an initial grip separation of 5 cm. The tensile test was performed with a strain rate of 0.08 mm/s. Toe compensation was performed for all measurements and tensile strength and elongation at break were measured utilising the Exponent connect Software v. 6.1.16.0 (Stable Micro Systems, Surrey, UK). The calculations were automatically adjusted for the measured thickness of the fiber mat by the software. Five specimens were analysed per fiber mat.

#### 2.3.3. Swelling Index

Swelling index of the nanofibers was tested using simulated wound fluid containing 5.84 g/L NaCl, 3.60 g/L NaHCO_3_, 0.30 g/L KCl, 0.37 g/L CaCl_2_·2H_2_O and 165 mL/L Albunorm^®^. This composition was slightly modified from a previously described simulated wound fluid, as we used human serum albumin (Albunorm^®^) instead of bovine albumin [46]. Fiber specimens were cut into 2 × 2 cm, weighed (W_I_, Table S1, Supplementary Material) and immersed in 2 mL simulated wound fluid for 5 min. When the specimen was picked up, excess fluid was removed using a tissue paper, and the fiber with absorbed fluid weighed again (W_A_). The experiment was repeated three times per formulation and the swelling index was calculated using the following Equation (1):

Swelling index (%) = (W_A_−W_I_)/W_I_ × 100 (1)

W_I_: Initial weight specimen
W_A_: Weight of specimen after swelling

#### 2.3.4. In Vitro Drug Release

Drug release from nanofibers containing CAM (βG-CHI-CAM-fiber, βG-CAM-fiber, CHI-CAM-fiber, Copol-CAM-fiber) was investigated using jacketed Franz diffusion cells (PermeGear, Inc., Hellertown, PA, USA) with a 5 mL acceptor chamber and a donor diffusion area of 0.64 cm^2^. A cellophane membrane (Max Bringmann KG, Wendelstein, Germany) was used to separate the acceptor and donor compartment as previously described [47]. The acceptor chamber contained 5 mL phosphate buffered saline (PBS) pH 7.4 (0.19 g/L KH_2_PO_4_, 2.98 g/L Na_2_HPO_4_·2H_2_O, 8 g/L NaCl) that was kept at a constant temperature of 32 °C. Round fiber samples with a diameter of 9 mm were cut, weighed (Table S2) and placed in the donor chamber. PBS (10 µL) was added to the fiber samples for hydration. As control, 300 µL of a CAM solution (0.3 mg/mL) in dH_2_O was used. A sample volume of 500 µL was withdrawn from the acceptor chamber after 10, 20, 30, 40, 60, 120, 180 and 360 min. The sample volume was instantly replaced with fresh PBS. CAM-content of the samples was quantified by UV-Vis spectrophotometry on a Spark^®^ multimode microplate reader (Tecan Trading AG, Männedorf, Switzerland) at λ = 278 nm.

### 2.4. Biological Tests

#### 2.4.1. In Vitro Cytotoxicity

The in vitro cytotoxicity of the nanofibers was tested in both human keratinocytes (HaCaT cells) and murine macrophages (RAW 264.7) using the Cell Counting Kit-8 (CCK-8, Sigma-Aldrich). HaCaT cells and RAW 264.7 cells were cultured in DMEM and RPMI-medium, respectively. Both media were supplemented with 10% (*v*/*v*) FBS and penicillin-streptomycin. Cells were seeded on 96 well plates (90 µL cell suspension/well; 1 × 10^5^ cells/mL) and incubated for 24 h at 37 °C in 5% CO_2_. Nanofibers were dissolved in 0.1% acetic acid (*w*/*w*) in Milli-Q-water at a concentration of 10 mg/mL and diluted with medium for further sample preparation. The cells were treated with 10 µL medium (control) or 10 µL nanofiber solutions (samples), corresponding to final fiber concentrations of 125, 250 and 1000 µg/mL. After further incubation for 24 h, CCK-8 reagent (10 µL) was added to each well and the plates were thereafter incubated for 4 h. Absorbance was measured at λ = 450 nm with a reference at λ = 650 nm using a Spark^®^ multimode microplate reader (Tecan Trading AG, Männedorf, Switzerland). Each sample-concentration was tested in triplicate. The relative cell viability was calculated using the following Equation (2):

Relative cell viability (%) = A_S_/A_C_ × 100 (2)

A_S_: Absorption of the samples
A_C_: Absorption of the control

#### 2.4.2. Antimicrobial Activity Testing Using the Disc Diffusion Assay

Antibacterial activity of nanofibers against *Escherichia coli* (*E. coli*) and *Staphylococcus aureus* (*S. aureus*), representing Gram-negative and Gram-positive bacteria, respectively, was determined using a modified disc diffusion assay, as described previously [48]. Bacterial suspensions were prepared in 0.9% NaCl with a turbidity of 0.5 McFarland. Bacterial suspensions were uniformly spread on Mueller-Hinton agar plates with a sterile cotton swab using an electric rotator. Nanofibers, cut into discs with a diameter of 6 mm, were adjusted in thickness to obtain the same CAM-content as in the positive control (6 mm 30 µg CAM disc, Thermo Fisher Scientific Inc. (Waltham, MA, USA). Nanofiber discs and a standard CAM positive-control were placed on the inoculated agar plate, and plates were incubated at 37 °C for 19 h. The zone of inhibition was measured using a scale. Three biological replicates were performed for each nanofiber and the results were presented as mean ± SD.

#### 2.4.3. Anti-Inflammatory Activity Testing

The anti-inflammatory activity of the nanofiber formulations was evaluated by measuring the NO production in LPS-stimulated murine macrophages (RAW 264.7) [49]. Samples were prepared as described in Section 2.4.1 (In vitro cytotoxicity test). Cells (5 × 10^5^ cells/mL) were seeded on 24-well plates (1 mL/well) and incubated at 37 °C in 5% CO_2_. After 24 h incubation, RPMI medium was replaced with 990 µL LPS-containing medium (1 µg/mL) and 10 µL of the dissolved formulations, corresponding to three different fiber concentrations (12.5, 25 and 100 µg/mL). Untreated cells were used as control. The cells were further incubated for 24 h. The NO production was measured by addition of Griess reagent (0.1% N-1-naphylenediamine dihydrochloride, 1% sulfanilamide, 2.5% phosphoric acid) in a ratio of 1:2 to samples from the cell supernatant. The absorption was measured on a Spark^®^ multimode microplate reader (Tecan Trading AG, Männedorf, Switzerland) at λ = 540 nm. The reduction in NO production was assumed to reflect the anti-inflammatory activity of the formulations on the cells. The anti-inflammatory activity was thus calculated as the NO production in cells treated with the formulations compared to untreated cells, using the following Equation (3):

NO production (%) = A_S_/A_C_ × 100 (3)

A_S_: Absorption of the samples
A_C_: Absorption of the control

### 2.5. Statistical Analysis

One-way ANOVA followed by a Tukey test using GraphPad Prism (Version 8.3.0, GraphPad Software, San Diego, CA, USA) was used to test for statistical significance. A *p*-value < 0.05 was considered statistically significant.

## 3. Results and Discussion

The first part of this study focused on the preparation of suitable polymer solutions for electrospinning using the needle-free electrospinning Nanospider^TM^ technology. Several variables, among them the solution composition and properties, ambient conditions and spinning settings, are known to influence the electrospinning process and nanofiber morphology [50]. We therefore decided to standardise ambient conditions, such as temperature and humidity, as well as electrospinning settings. Finally, the total polymer concentration was standardised to 2.5% (*w*/*w*). The solvent system, regarded as suitable for solubilising all components, as well as being eco-friendly, consisted of ethanol, acetic acid and water. The standardisation of the experimental setup was performed in preliminary experiments (results not shown).

### 3.1. Effect of Polymer Solution Properties on Nanofiber Morphology

Important features of polymer solutions used in electrospinning are the surface tension, conductivity and viscosity, as these characteristics are known to influence both the spinning process and quality of the formed fibers [11]. The polymer needs to have a certain chain entanglement, represented by the polymer solutions viscosity, to avoid jet breakup during the spinning process [11]. On the other hand, a too high viscosity has previously been shown to cause problems for electrospinning of solutions in the Nanospider^TM^, since a high viscosity might limit the gravidity-dependant feeding rate of the electrospinning wire [23]. In addition, the surface tension and conductivity play an important role in Taylor cone formation and fiber elongation [39]. The influence of the solutions properties on the needle-spinning process has been widely studied [50,51,52,53]. However, the solutions optimal features when using the Nanospider^TM^ system might be different. Thus, we focused on their effect on the morphology of the obtained nanofibers.

The characteristics of the polymer solutions are summarised in Table 2. Solutions containing CHI had a higher pH and a higher conductivity, both attributed to the protonation of the amino groups in CHI in acidic media [53,54]. Furthermore, the polycationic nature of CHI in acidic medium is known to give an enlarged chain conformation [55]. This might explain the higher viscosity of CHI-containing solutions compared to solutions containing only the neutral polymers (βG, PEO, HPMC). None of the solution ingredients affected the surface tension of the polymer solutions, which was between 26.4 and 27.9 mN/m for all solutions. This relatively low surface tension can be explained by the relative high ethanol content in all polymer solutions (60% (*w*/*w*)). The addition of CAM did not affect the solution properties (Table 2).

All polymer solutions were spun applying the same settings (described in Section 2.2.3), and all solutions successfully formed a white nanofibrous mat on the substrate. Thus, the selected polymer solution compositions and the electrospinning setup were both judged proper. The viscosity of all solutions was within the range that allows a smooth feeding of the electrospinning wire. Macroscopically, CHI-containing nanofibers (βG-CHI-nf, βG-CHI-CAM-nf, CHI-nf and CHI-CAM-nf) had a smooth surface while βG-containing nanofibers without CHI (βG-nf and βG-CAM-nf) had a rougher surface. The nanofibers containing only the co-polymers (Copol-nf and Copol-CAM-nf) were more brittle and difficult to remove from the substrate compared to the other nanofibers.

**Figure 1:**
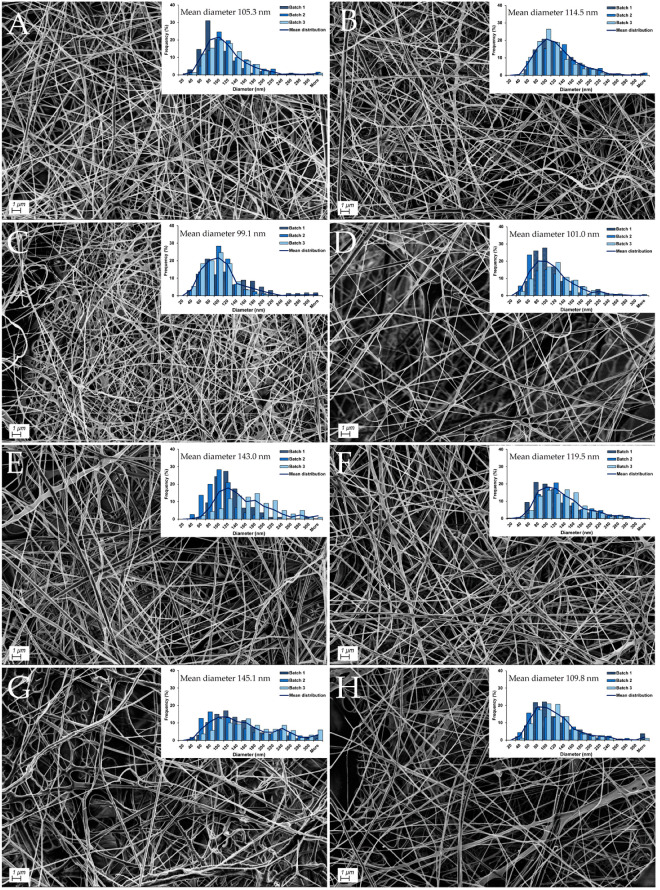
Representative SEM images for each nanofiber formulation. Fiber diameter distribution was determined by measurement of 300 single-fiber diameters for each batch. The overall mean diameter can be found above the diameter distribution (*n* = 3). (**A**): βG-CHI-nf, (**B**): βG-CHI-CAM-nf, (**C**): βG-nf, (**D**): βG-CAM-nf, (**E**): CHI-nf, (**F**): CHI-CAM-nf, (**G**): Copol-nf, (**H**): Copol-CAM-nf. Abbreviations: βG (β-glucan), CHI (chitosan), Copol (co-polymers: polyethylene oxide and hydroxypropylmethylcellulose), CAM (chloramphenicol), nf (nanofiber).

The morphology and diameter distribution of CAM-free and CAM-containing nanofibers obtained from the different polymer solutions is shown in Figure 1. The SEM pictures confirmed that all solutions were able to form nanofibers with a final concentration of 2.5% (*w*/*w*) dry material. Although it has been reported previously that a surface tension above 42 mN/m was needed for the formation of beadless PEO/Curdlan fibers by needle-electrospinning [56], we experienced that polymer solutions with a low surface tension ranging from 26.4 to 27.9 mN/m resulted in beadless fiber formation when spun by the Nanospider^TM^. The lower surface tension required in needle-free electrospinning is in accordance with previously published data: Ramakrishnan et al. examined the effect of solution parameters in needle-free electrospinning and found that a low surface tension (32 mN/m) improved the quality of PEO-nanofibers [39].

All nanofibers had a relatively small mean fiber diameter, ranging from 99 to 150 nm (Figure 1). The fiber diameter did not vary significantly between the different nanofiber formulations. Although one batch from each of the two formulations βG-CAM-nf and CHI-CAM-nf was spun with a humidity outside the set limitations of 24 to 29%, these nanofibers did not show any changes in the diameter distribution (Figure 1) and both batches were therefore included in the study. No differences in fiber morphology or diameter could be seen upon addition of 1% (*w*/*w*) CAM. This was expected since polymer solutions with and without CAM showed similar properties (Table 2). This diameter range was in agreement with literature: as the same diameter range has been reported by others using the same Nanospider^TM^ technology: CHI-nanofibers with a mean diameter of 110 nm [24] and βG-containing nanofibers with a mean diameter of between 110 and 180 nm [23].

### 3.2. Mechanical Properties of Nanofibers

Easy management and handling of the nanofibers in a clinical setting requires a certain mechanical strength. Thus, their tensile strength and elongation at break were examined using a texture analyzer. The tensile strength is the force needed to rupture the fibrous mats, whereas the elongation at break is the strain the fibrous mats had when ruptured. Since these parameters are correlating with their thickness, the thickness was examined using a micrometre. The results are summarized in Table 3.

All nanofibrous mats had a mean thickness ranging from 35 to 86 µm and were thinner on the edges compared to the middle of the nanofibrous mats. This can be explained by the pattern of fiber deposition provided by the Nanospider^TM^ during the electrospinning process. CHI-containing nanofibrous mats were thinner compared to CHI-free nanofibrous mats (Table 3). During the electrospinning process, it was noticed that CHI-containing nanofibrous mats exhibited a wider distribution of fibers and thus covered a wider area on the substrate compared to the nanofibers without CHI. This might explain the lower thickness of the CHI nanofibers. The difference in thickness correlates with the differences in solution properties of CHI containing solutions compared to solutions without CHI (Table 2). CHI containing solutions showed a significantly higher conductivity and viscosity. These properties are known to influence the electrospinning process and might have affected the distribution of the fibers during electrospinning.

Although the mean tensile strength of the nanofibers varied between 9.2 MPa (βG-CAM-nf) and 21.8 MPa (βG-nf), no significant difference in tensile strength could be seen between the different formulations due to the high standard deviations (Table 3). The addition of CAM into the fibers did not affect the tensile strength significantly. In contrary to this, the elongation at break was significantly reduced for fibers containing βG and no CHI (βG-nf and βG-CAM-nf) compared to the fibers formed of only co-polymers (Copol-nf and Copol-CAM-nf). For comparison, the tensile strength for human skin collected from the back has been reported to be 21.6 ± 8.4 MPa [57]. In the same study, an elongation at break of 54 ± 17% was reported. This implies that the dry nanofibers have a similar tensile strength but a poorer elongation at break compared to the human skin, as the elongation at break of the nanofibers in this study was only between 3.5 and 8.9%. We judged our nanofibers to have a sufficiently high mechanical strength for physical handling in a clinical setting. However, for application within the wound bed, secondary backing materials might be beneficial to improve wound protection and exudate absorption.

### 3.3. Swelling Properties of Nanofibers

It is critical to maintain a moisture balance in the wound, both for an optimal wound healing, and to limit the scar formation [58,59]. Polymers in the nanofibers will start to hydrate upon exposure to fluids, and further expand through fluid absorption, forming a gel. The absorption behaviour of a nanofibrous dressing is usually given as the swelling index. The swelling index of nanofibers was calculated from the difference in nanofiber-weight before and after immersion in wound fluid, as given in equation (2). The results are given in Table 4. Fibers without CHI (βG-CAM-nf, and Copol-CAM-nf) rapidly turned into a disintegrated hydrogel when added to the fluid and they could therefore not be separated from the wound fluid. Thus, no weight after wetting or swelling index could be reported for these formulations. The disintegration of βG-CAM-nf and Copol-CAM-nf in the fluid indicates furthermore that their fibrous structure was not maintained. A possible strategy to better maintain the fibrous structure would be by crosslinking of the nanofibrous mats since crosslinking is known to enhance the stability and resistance to chemical degradation of a scaffold [60].

In contrast, nanofibers containing CHI (βG-CHI-CAM-nf, CHI-CAM-nf) showed a higher stability upon contact to fluid. These nanofibers could therefore be withdrawn from the media after 5 min. These fibers were found to have a high swelling index between 779 and 1055% (Table 4). As swelling indices of the same magnitude have been reported previously for nanofibers containing some of the same polymers, this result confirms the hydrophilic and hydroscopic nature of the polymers used. CHI-PEO fibers were previously found to have a swelling index of 1132% [61] and βG-containing nanofibers with HPMC and PEO as co-polymers had a swelling index of 1287 ± 109% in a previous study [23].

A high swelling index is linked to several beneficial features of a wound dressing, as a wound dressing should instantly attach to the wound and adapt to its shape to assure a tight barrier, protecting against bacterial invasion and allowing the best possible tissue contact [62]. In addition, the dressing should provide a moist environment and be able to take up excess exudate to avoid maceration of the wound bed [63].

All nanofibers in this study swelled to a high degree and formed a transparent gel, suggesting that they will immediately attach to a wound, adjust to its shape and allow the examination of the wound without the need to remove the dressing due to its transparency.

### 3.4. In Vitro Release of Chloramphenicol

**Figure 2:**
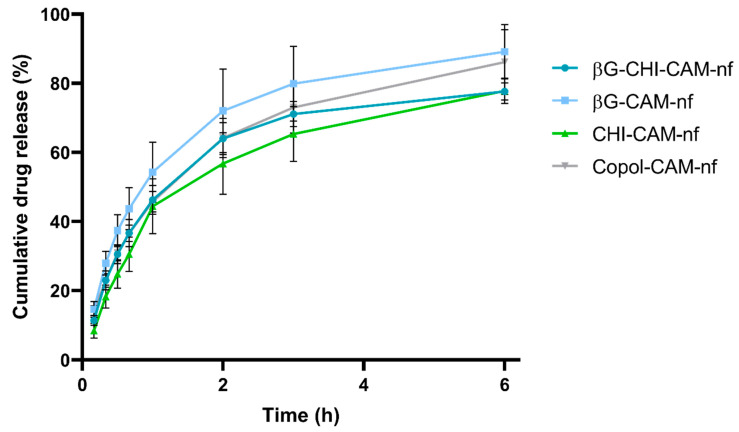
Cumulative release (%) of chloramphenicol from nanofibers during a 6 h test-period in a Franz diffusion setup. Abbreviations: βG (β-glucan), CHI (chitosan), Copol (co-polymers: polyethylene oxide and hydroxypropylmethylcellulose), CAM (chloramphenicol), nf (nanofibers). Results are presented as mean ± SD (*n* = 3).

The release kinetics of the antibiotic ingredient, in this case CAM, will affect the antimicrobial effectivity of the dressing. For successful treatment of infections, a therapeutically active concentration of the antibiotic agent(s) needs to be present at the wound site. The in vitro release of CAM from nanofibers was tested for 6 h in a Franz diffusion setup. The CAM-release is shown in Figure 2, displayed as the cumulative release (%). All nanofibers had a burst release, with 55 to 70% of the CAM being released within the first 2 h, and a maximum CAM-release of between 77 and 89% reached already after 6 h (Figure 2). In the treatment of infections, an initial burst release of antibiotics is expected to be beneficial, since it will help to rapidly reach the needed/effective concentration of the antibiotic on the wounded site, and enable a rapid impeding of bacterial growth and further spreading [64].

The burst drug release from nanofibers was expected, taking into account (i) the thin fiber diameter of between 99 to 150 nm (Figure 1), (ii) the high swelling index of between 800 and 1400% (Table 4) and (iii) the hydrophilicity of the polymers, all of which are known to influence the release from nanofibers [65]. A faster swelling reflects the faster penetration of water molecules into the fibrous structure. This leads to a more rapid dissolution of the drug molecules. With increased time, erosion of the fibers will start, freeing the last drug molecules [66]. A small fiber diameter, as observed in this study (Figure 1), will also give a higher surface area to volume ratio and shorter diffusion distance, leading to a faster drug release. Although CHI-containing nanofibers showed improved mechanical stability upon contact with water, they had a very high swelling index, which explains the similar drug release profile from all nanofiber formulations.

### 3.5. Cytotoxicity Testing

**Figure 3:**
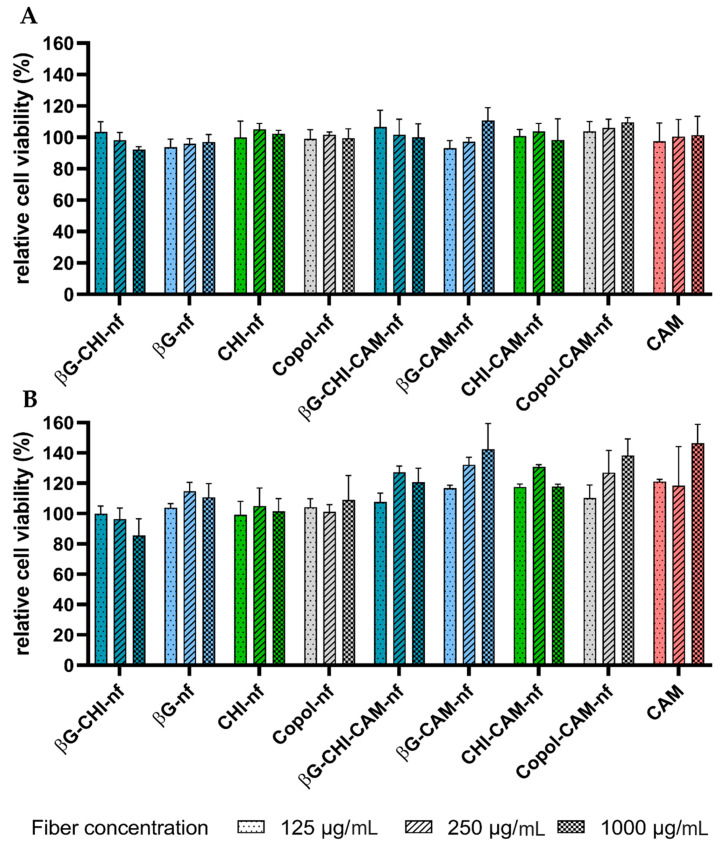
Relative cell viability (%) of (**A**) HaCaT cells and (**B**) macrophages (RAW 264.7) after 24 h incubation at 37 °C and exposure to nanofibers (dissolved in concentrations of 125, 250 and 1000 µg/mL) and chloramphenicol (CAM) (in concentrations of 1.25, 2.5 and 10 µg/mL). Results are presented as mean ± SD (*n* = 3). Abbreviations: βG (β-glucan), CHI (chitosan), Copol (co-polymers: polyethylene oxide and hydroxypropylmethylcellulose), CAM (chloramphenicol), nf (nanofiber).

An ideal dressing should, amongst other features, be biodegradable and biocompatible [9]. The cytotoxicity of the nanofibers in this study was tested on keratinocytes (HaCaT cells) and macrophages (RAW 264.7). These two cell lines were selected since they are central actors in the wound healing process. Keratinocytes are not only the primary cells in the epidermis, but also responsible for several immune functions during wound healing [67]. Macrophages are regulators of the healing process in all stages of the healing process and contribute to, e.g., cleaning and restoration of the wound [68]. A formulation that is reducing their viability might therefore hinder the wound healing process. As shown in Figure 3, both HaCaT cells and RAW 264.7 cells maintained a cell viability over 80% in presence of the different nanofiber formulations in all the tested concentrations. Since a reduction in cell viability lower than 30% is considered to indicate that the formulation is non-toxic [69], the nanofibers in this study were concluded to show no cytotoxicity in the tested cell lines.

### 3.6. Antimicrobial Activity

Infections are one of the leading causes for death after burn injuries; therefore, the use of a dressing that has antimicrobial activity is favourable [2]. We fabricated nanofibers with CHI, a polymer that has previously been shown to have antimicrobial activity, and incorporated the antibiotic CAM for local antibiotic delivery. For successful treatment of wound infections, an antibiotic concentration above the minimal inhibition concentration (MIC) must be reached. For these reasons, we assessed the antimicrobial activity of the nanofibers applying a modified disc diffusion assay with *E. coli* and *S. aureus* as representative gram-negative and gram-positive bacteria, respectively. Both these pathogens are commonly found in infected burn wounds [70].

**Figure 4:**
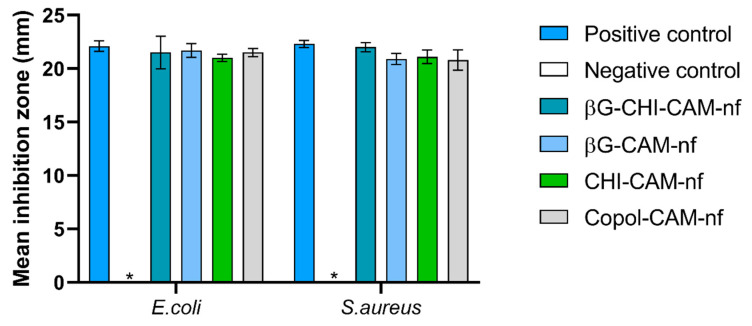
Antibacterial activity of chloramphenicol-containing nanofibers (containing 30 µg of chloramphenicol per fiber) compared to a standard 30 µg chloramphenicol disc as positive control and no-CAM containing nanofibers as negative control (*). Results are expressed as the mean inhibition zone (mm) ± SD (*n* = 3). Abbreviations: βG (β-glucan), CHI (chitosan), Copol (co-polymers: polyethylene oxide and hydroxypropylmethylcellulose), CAM (chloramphenicol), nf (nanofiber).

The mean inhibition zones of CAM-containing nanofibers were compared to a 30 µg CAM standard disc as a positive control. Our results showed no bacterial inhibition from any of the nanofibers without CAM, neither for *E. coli* nor for *S. aureus* (Figure 4). We, therefore, defined them as “negative control” (Figure 4 and Figure S1). All CAM-containing nanofibers showed an antimicrobial effect on both *E.coli* and *S. aureus* similar to the positive control (Figure 4). This proves that the antimicrobial activity of CAM is maintained in the nanofibers and that CAM was intact and evenly distributed in the nanofibers after electrospinning.

CHI is known to have antimicrobial properties and Abid et al. reported that CHI-PEO fibers showed zones of inhibition of 22.3 ± 3.0 nm and 14.7 ± 1.36 nm (over 24 h) for *E. coli* and *S. aureus*, respectively [71]. However, no such activity was observed in this study. This might be explained by the neutral pH of the gel that is formed from the nanofibers when they are placed on the agar plate, since an acidic pH is needed to support the proposed mechanism for CHI’s antimicrobial properties that depend on the interaction between the negatively charged bacterial membrane and the positively charged CHI [72]. Our nanofibers were spun with a low concentration of acetic acid (3% (*w*/*w*)) and most of the acetic acid evaporated during the electrospinning process, resulting in fibers with a neutral pH when applied on the agar plate (pH measurements not shown). This implies that CHI is not protonated, and thus does not have the aforementioned antimicrobial effect. Similar observations have been described previously, for CHI films containing 2% (*w*/*v*) CHI, prepared in 2% (*v*/*v*) acetic acid solution [73]. Here, no antimicrobial activity was observed form the CHI-containing films, whereas CHI-solutions led to significant inhibition of *E. coli*, *S. aureus* and *S. epidermidis*. On the contrary, Abid et al. showed an antimicrobial effect from fibers spun with a solvent containing 50% acetic acid [71]. Thus, a higher residue of acetic acid in the fibers might maintain the antimicrobial activity of CHI. Although CHI did not improve the antimicrobial properties in our fibers, the mechanical strength upon contact with water was improved by including CHI (Table 4). Thus, all formulations, also the CHI-containing ones, were included in the further assessment, which involved the anti-inflammatory activity of the nanofibers.

### 3.7. Anti-Inflammatory Activity

The occurrence of severe burns in combination with the following surgical actions can lead to an uncontrolled inflammatory reaction [2]. Although an initial inflammatory response is needed for the healing process, an uncontrolled and persistent reaction can lead to organ dysfunction and death [2]. Macrophages play an important role in the wound healing process, also in the inflammation process, and are therefore interesting to target in wound therapy [74].

**Figure 5:**
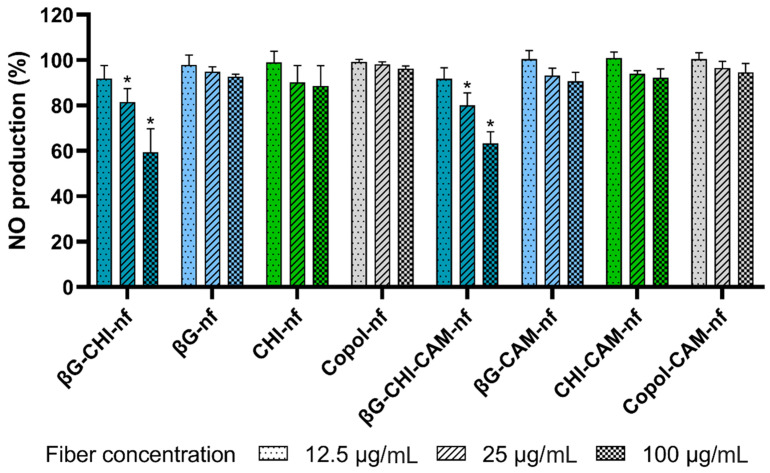
NO production (%) of LPS-induced macrophages (RAW 264.7 cells) after 24 h exposure to nanofibers in three different concentrations (12.5, 25 and 100 µg/mL) compared to untreated cells. Abbreviations: βG (β-glucan), CHI (chitosan), Copol (co-polymers: polyethylene oxide and hydroxypropylmethylcellulose), CAM (chloramphenicol), nf (nanofiber). Results are presented as mean ± SD (*n* = 3). Formulations marked with * are statistically significant (*p* < 0.05) compared to untreated LPS-stimulated macrophages.

Anti-inflammatory activity of both βG and CHI in macrophages has been reported previously [75,76]. The anti-inflammatory effect from the formulations on macrophages can be measured as the reduction in NO-production (%) in macrophages after treatment with LPS compared to untreated cells as negative control (Figure 5).

We found a significant concentration-dependent inhibition of NO production for nanofibers containing both βG and CHI (βG-CHI-nf and βG-CHI-CAM-nf). At the same time, nanofibers formed from only the co-polymers did not influence the NO production and showed no anti-inflammatory activity. Nanofibers containing only one of the active polymers (βG or CHI) had just a slight and not significant reduction in NO production with increasing concentration (Figure 5). The low anti-inflammatory activity of nanofibers containing only one of the active ingredients might be due to the low fiber concentration tested in this study (12.5, 25 and 100 µg/mL). If higher nanofiber concentrations, e.g., in the concentration range applied in the cytotoxicity testing (125, 250 and 1000 µg/mL, Figure 3) would have been applied, a more pronounced effect might have been observed. However, we were able to demonstrate that the nanofibers in concentrations well below toxic concentration levels have an anti-inflammatory effect.

Since the anti-inflammatory effect of nanofibers containing both active polymers (βG-CHI-nf and βG-CHI-CAM-nf) was higher than the combined decrease in NO production from the nanofibers containing only βG (βG-nf and βG-CAM-nf) or CHI (CHI-nf and CHI-CAM-nf), a synergistic effect is suggested when combining these two active biopolymers in the same dressing. It seems like the low concentrations of βG and CHI tested separately did not affect the macrophages while the combination of the two ingredients led to a concentration that was high enough to modulate the anti-inflammatory response of macrophages, thus leading to a reduction in NO production. The production of NO is an indication of inflammation in macrophages [75]. NO has an important function in inflammation and its dysregulation is connected to the development of a multiple organ failure after burn injuries [77]. The exposure of burn wounds to dressings containing anti-inflammatory polymers such as βG and CHI could therefore be effective for their treatment.

In this study, we fabricated nanofibrous wound dressings with three different active ingredients, βG, CHI and CAM. The formulation combining all three active ingredients (βG-CHI-CAM-nf) showed anti-inflammatory properties (Figure 5) as well as antimicrobial properties (Figure 4). CHI did not enhance the antimicrobial properties of the dressing. However, the combination of CHI with βG in one dressing led to improved anti-inflammatory properties even at low concentrations and improved mechanical properties of the dressing.

## 4. Conclusions

Nanofibrous wound dressings comprising the bioactive polymers; soluble beta-glucan (SBG^®^) and/or chitosan, and with or without 1% (*w*/*w*) chloramphenicol, were successfully prepared by needle-free electrospinning, and their morphology examined by FE-SEM. All formulations formed uniform nanofibers with a diameter of approximately 100 nm. Among the tested formulations, was the formulation containing all three active ingredients; beta-glucan, chitosan and chloramphenicol, judged the most promising, supported by the in vitro anti-inflammatory activity observed in macrophages, which was higher for this formulation compared to the other formulations. Furthermore, the antimicrobial activity of chloramphenicol was well-maintained in all nanofibers, demonstrated by the disc diffusion assay with two bacterial strains: *E. coli* and *S. aureus*. These characteristics, as well as the in vitro biocompatibility of the formulation in HaCaT and RAW 264.7 cell lines, make these dressings promising for infected wounds and wounds with a high inflammatory reaction.

## Figures and Tables

**Table 1:** Composition (% (*w*/*w*)) of the dry material in the polymer solutions.

Polymer Solutions	Ingredients				
	βG (%)	CHI (%)	PEO (%)	HPMC (%)	CAM (%)
βG-CHI-sol	20.0	20.0	30.0	30.0	-
βG-sol	20.0	-	40.0	40.0	-
CHI-sol	-	20.0	40.0	40.0	-
Copol-sol	-	-	50.0	50.0	-
βG-CHI-CAM-sol	20.0	20.0	29.5	29.5	1.0
βG-CAM-sol	20.0	-	39.5	39.5	1.0
CHI-CAM-sol	-	20.0	39.5	39.5	1.0
Copol-CAM-sol	-	-	49.5	49.5	1.0

Abbreviations: βG (β-glucan), CHI (chitosan), Copol (co-polymers: polyethylene oxide and hydroxypropylmethylcellulose), CAM (chloramphenicol), sol (solution).

**Table 2:** Polymer solution characteristics. Results are presented as mean ± SD (*n* = 3).

Polymer Solutions	Solution Characteristics			
	pH	Surface Tension (mN/m)	Conductivity (µS/cm)	Viscosity (Pa·s)
βG-CHI-sol	4.62 ± 0.03	27.4 ± 0.5	143.2 ± 18.2	2.37 ± 0.52
βG-sol	3.89 ± 0.01	26.6 ± 0.4	64.5 ± 1.6	1.23 ± 0.15
CHI-sol	4.57 ± 0.01	26.9 ± 0.5	138.4 ± 3.3	2.30 ± 0.02
Copol-sol	3.91 ± 0.09	26.4 ± 0.4	71.0 ± 1.2	1.09 ± 0.08
βG-CHI-CAM-sol	4.53 ± 0.03	27.9 ± 0.4	143.3 ± 11.5	2.55 ± 0.28
βG-CAM-sol	3.78 ± 0.06	26.8 ± 0.8	65.1 ± 2.0	1.17 ± 0.15
CHI-CAM-sol	4.52 ± 0.03	27.6 ± 1.0	148.9 ± 7.5	2.34 ± 0.25
Copol-CAM-sol	3.92 ± 0.11	27.1 ± 1.0	70.1 ± 1.6	1.09 ± 0.20

Abbreviations: βG (β-glucan), CHI (chitosan), Copol (co-polymers: polyethylene oxide and hydroxypropylmethylcellulose), CAM (chloramphenicol), sol (solution).

**Table 3:** Mechanical characteristics of nanofibers. Results are presented as mean ± SD (*n* = 3).

Formulation	Nanofiber Characteristics and Mechanical Properties		
	Thickness (µm)	Tensile Strength (MPa)	Elongation at Break (%)
βG-CHI-nf	35.1 ± 8.7	21.4 ± 18.7	3.5 ± 0.8
βG-nf	71.7 ± 11.8	21.8 ± 13.6	4.8 ± 1.4
CHI-nf	47.3 ± 5.1	17.1 ± 3.7	7.3 ± 0.9
Copol-nf	64.3 ± 7.9	21.2 ± 12.0	8.5 ± 0.6
βG-CHI-CAM-nf	38.0 ± 1.2	12.2 ± 7.6	5.4 ± 2.1
βG-CAM-nf	85.8 ± 11.2	9.2 ± 4.2	3.9 ± 0.7
CHI-CAM-nf	55.5 ± 7.2	20.8 ± 6.2	8.9 ± 1.8
Cop-CAM-nf	72.7 ± 9.0	15.7 ± 4.3	6.7 ± 0.9

Abbreviations: βG (β-glucan), CHI (chitosan), Copol (co-polymers: polyethylene oxide and hydroxypropylmethylcellulose), CAM (chloramphenicol), nf (nanofiber).

**Table 4:** Swelling index of nanofibers containing chloramphenicol. Results are presented as mean ± SD (*n* = 3). The swelling index of nanofibers marked with * could not be measured.

Formulation	Swelling Index (%)
βG-CHI-CAM-nf	1055 ± 318
βG-CAM-nf *	-
CHI-CAM-nf	779 ± 242
Cop-CAM-nf *	-

Abbreviations: βG (β-glucan), CHI (chitosan), Copol (co-polymers: polyethylene oxide and hydroxypropylmethylcellulose), CAM (chloramphenicol), nf (nanofiber).

## Data Availability

Data is contained within the article or supplementary material.

## Supplementary Materials

The following are available online at https://www.mdpi.com/article/10.3390/pharmaceutics13091527/s1, Figure S1: Antibacterial activity of nanofibers, Table S1: Weight of the nanofibrous mats used for the swelling index testing, Table S2: Weight of the nanofibrous mats used for the drug release testing.

## References

[1] Sganga G., Pea F., Aloj D., Corcione S., Pierangeli M., Stefani S., Rossolini G.M., Menichetti F. (2020). Acute wound infections management: The ’Don’ts’ from a multidisciplinary expert panel. Expert Rev. Anti-Infect. Ther..

[2] Jeschke M.G., van Baar M.E., Choudhry M.A., Chung K.K., Gibran N.S., Logsetty S. (2020). Burn injury. Nat. Rev. Dis. Primers.

[3] Campoccia D., Montanaro L., Speziale P., Arciola C.R. (2010). Antibiotic-loaded biomaterials and the risks for the spread of antibiotic resistance following their prophylactic and therapeutic clinical use. Biomaterials.

[4] Luraghi A., Peri F., Moroni L. (2021). Electrospinning for drug delivery applications: A review. J. Control Release.

[5] Lorenzo D. (2019). Chloramphenicol Resurrected: A Journey from Antibiotic Resistance in Eye Infections to Biofilm and Ocular Microbiota. Microorganisms.

[6] Falagas M.E., Grammatikos A.P., Michalopoulos A. (2008). Potential of old-generation antibiotics to address current need for new antibiotics. Expert Rev. Anti-Infect. Ther..

[7] Andaluz-Scher L., Medow N.B. (2020). Chloramphenicol Eye Drops: An Old Dog in a New House. Ophthalmology.

[8] Takada S., Fujiwara S., Inoue T., Kataoka Y., Hadano Y., Matsumoto K., Morino K., Shimizu T. (2016). Meningococcemia in Adults: A Review of the Literature. Intern. Med..

[9] Souto E.B., Ribeiro A.F., Ferreira M.I., Teixeira M.C., Shimojo A.A.M., Soriano J.L., Naveros B.C., Durazzo A., Lucarini M., Souto S.B. (2020). New Nanotechnologies for the Treatment and Repair of Skin Burns Infections. Int. J. Mol. Sci..

[10] Maleki Dizaj S., Sharifi S., Jahangiri A. (2019). Electrospun nanofibers as versatile platform in antimicrobial delivery: Current state and perspectives. Pharm. Dev. Technol..

[11] Juncos Bombin A.D., Dunne N.J., McCarthy H.O. (2020). Electrospinning of natural polymers for the production of nanofibres for wound healing applications. Mater. Sci. Eng. C.

[12] Dubsky M., Kubinova S., Sirc J., Voska L., Zajicek R., Zajicova A., Lesny P., Jirkovska A., Michalek J., Munzarova M. (2012). Nanofibers prepared by needleless electrospinning technology as scaffolds for wound healing. J. Mater. Sci. Mater. Med..

[13] Pelipenko J., Kocbek P., Govedarica B., Rosic R., Baumgartner S., Kristl J. (2013). The topography of electrospun nanofibers and its impact on the growth and mobility of keratinocytes. Eur. J. Pharm. Biopharm..

[14] Doostmohammadi M., Forootanfar H., Ramakrishna S. (2020). Regenerative medicine and drug delivery: Progress via electrospun biomaterials. Mater. Sci. Eng. C.

[15] Memic A., Abudula T., Mohammed H.S., Joshi Navare K., Colombani T., Bencherif S.A. (2019). Latest Progress in Electrospun Nanofibers for Wound Healing Applications. ACS Appl. Bio Mater..

[16] Sill T.J., von Recum H.A. (2008). Electrospinning: Applications in drug delivery and tissue engineering. Biomaterials.

[17] Geng Y., Zhou F., Williams G.R. (2021). Developing and scaling up fast-dissolving electrospun formulations based on poly(vinylpyrrolidone) and ketoprofen. J. Drug Deliv. Sci. Technol..

[18] Quan Z., Wang Y., Zu Y., Qin X., Yu J. (2021). A rotary spinneret for high output of electrospun fibers with bimodal distribution. Eur. Polym. J..

[19] Vass P., Szabo E., Domokos A., Hirsch E., Galata D., Farkas B., Demuth B., Andersen S.K., Vigh T., Verreck G. (2020). Scale-up of electrospinning technology: Applications in the pharmaceutical industry. Wiley Interdiscip. Rev. Nanomed. Nanobiotechnol..

[20] Molnar K., Nagy Z.K. (2016). Corona-electrospinning: Needleless method for high-throughput continuous nanofiber production. Eur. Polym. J..

[21] Omer S., Forgách L., Zelkó R., Sebe I. (2021). Scale-up of Electrospinning: Market Overview of Products and Devices for Pharmaceutical and Biomedical Purposes. Pharmaceutics.

[22] Yalcinkaya F. (2017). Preparation of various nanofiber layers using wire electrospinning system. Arab. J. Chem..

[23] Grip J., Engstad R.E., Skjaeveland I., Škalko-Basnet N., Isaksson J., Basnet P., Holsæter A.M. (2018). Beta-glucan-loaded nanofiber dressing improves wound healing in diabetic mice. Eur. J. Pharm. Sci..

[24] Zhang S., Xu Z., Wen X., Wei C. (2021). A nano chitosan membrane barrier prepared via Nanospider technology with non-toxic solvent for peritoneal adhesions’ prevention. J. Biomater. Appl..

[25] El-Newehy M.H., Al-Deyab S.S., Kenawy E.-R., Abdel-Megeed A. (2011). Nanospider Technology for the Production of Nylon-6 Nanofibers for Biomedical Applications. J. Nanomater..

[26] Partheniadis I., Nikolakakis I., Laidmäe I., Heinämäki J. (2020). A Mini-Review: Needleless Electrospinning of Nanofibers for Pharmaceutical and Biomedical Applications. Processes.

[27] Li Z., Mei S., Dong Y., She F., Li Y., Li P., Kong L. (2020). Functional Nanofibrous Biomaterials of Tailored Structures for Drug Delivery-A Critical Review. Pharmaceutics.

[28] Abrigo M., Kingshott P., McArthur S.L. (2015). Electrospun Polystyrene Fiber Diameter Influencing Bacterial Attachment, Proliferation, and Growth. ACS Appl. Mater. Interfaces.

[29] Boateng J., Catanzano O. (2015). Advanced Therapeutic Dressings for Effective Wound Healing-A Review. J. Pharm. Sci..

[30] Majtan J., Jesenak M. (2018). β-Glucans: Multi-Functional Modulator of Wound Healing. Molecules.

[31] Engstad R.E., Robertsen B. (1994). Specificity of a β-glucan receptor on macrophages from Atlantic salmon (*Salmo salar* L.). Dev. Comp. Immunol..

[32] Engstad C.S., Engstad R.E., Olsen J., Østerud B. (2002). The effect of soluble β-1,3-glucan and lipopolysaccharide on cytokine production and coagulation activation in whole blood. Int. Immunopharmacol..

[33] Zykova S.N., Balandina K.A., Vorokhobina N.V., Kuznetsova A.V., Engstad R., Zykova T.A. (2014). Macrophage stimulating agent soluble yeast β-1,3/1,6-glucan as a topical treatment of diabetic foot and leg ulcers: A randomized, double blind, placebo-controlled phase II study. J. Diabetes Investig..

[34] Gianino E., Miller C., Gilmore J. (2018). Smart Wound Dressings for Diabetic Chronic Wounds. Bioengineering.

[35] Hurler J., Berg O.A., Skar M., Conradi A.H., Johnsen P.J., Škalko-Basnet N. (2012). Improved Burns Therapy: Liposomes-in-Hydrogel Delivery System for Mupirocin. J. Pharm. Sci..

[36] Hemmingsen L.M., Giordani B., Pettersen A.K., Vitali B., Basnet P., Škalko-Basnet N. (2021). Liposomes-in-chitosan hydrogel boosts potential of chlorhexidine in biofilm eradication in vitro. Carbohydr. Polym..

[37] Deng L., Taxipalati M., Zhang A., Que F., Wei H., Feng F., Zhang H. (2018). Electrospun Chitosan/Poly(ethylene oxide)/Lauric Arginate Nanofibrous Film with Enhanced Antimicrobial Activity. J. Agric. Food Chem..

[38] Barzegar S., Zare M.R., Shojaei F., Zareshahrabadi Z., Koohi-Hosseinabadi O., Saharkhiz M.J., Iraji A., Zomorodian K., Khorram M. (2021). Core-shell chitosan/PVA-based nanofibrous scaffolds loaded with Satureja mutica or Oliveria decumbens essential oils as enhanced antimicrobial wound dressing. Int. J. Pharm..

[39] Ramakrishnan R., Gimbun J., Ramakrishnan P., Ranganathan B., Reddy S.M.M., Shanmugam G. (2019). Effect of Solution Properties and Operating Parameters on Needleless Electrospinning of Poly (Ethylene Oxide) Nanofibers Loaded with Bovine Serum Albumin. Curr. Drug Deliv..

[40] Pakravan M., Heuzey M.-C., Ajji A. (2011). A fundamental study of chitosan/PEO electrospinning. Polymer.

[41] Mašková E., Kubová K., Raimi-Abraham B.T., Vllasaliu D., Vohlídalová E., Turánek J., Mašek J. (2020). Hypromellose–A traditional pharmaceutical excipient with modern applications in oral and oromucosal drug delivery. J. Control. Release.

[42] Stie M.B., Jones M., Sorensen H.O., Jacobsen J., Chronakis I.S., Nielsen H.M. (2019). Acids ’generally recognized as safe’ affect morphology and biocompatibility of electrospun chitosan/polyethylene oxide nanofibers. Carbohydr. Polym..

[43] Ho D.L., Hammouda B., Kline S.R., Chen W.-R. (2006). Unusual Phase Behavior in Mixtures of Poly(ethylene oxide) and Ethyl Alcohol. J. Polym. Sci. B Polym. Phys..

[44] Rasband W.S. (1997–2018). ImageJ.

[45] ASTM D882-18 (2018). Standard Test Method for Tensile Properties of Thin Plastic Sheeting.

[46] Bradford C., Freeman R., Percival S.L. (2009). In Vitro Study of Sustained Antimicrobial Activity of a New Silver Alginate Dressing. J. Am. Col. Certif. Wound. Spec..

[47] Joraholmen M.W., Vanic Z., Tho I., Škalko-Basnet N. (2014). Chitosan-coated liposomes for topical vaginal therapy: Assuring localized drug effect. Int. J. Pharm..

[48] Amiri N., Ajami S., Shahroodi A., Jannatabadi N., Amiri Darban S., Fazly Bazzaz B.S., Pishavar E., Kalalinia F., Movaffagh J. (2020). Teicoplanin-loaded chitosan-PEO nanofibers for local antibiotic delivery and wound healing. Int. J. Biol. Macromol..

[49] Cauzzo J., Nystad M., Holsæter A.M., Basnet P., Škalko-Basnet N. (2020). Following the Fate of Dye-Containing Liposomes In Vitro. Int. J. Mol. Sci..

[50] Haider A., Haider S., Kang I.-K. (2018). A comprehensive review summarizing the effect of electrospinning parameters and potential applications of nanofibers in biomedical and biotechnology. Arab. J. Chem..

[51] Akinalan Balik B., Argin S. (2019). Role of rheology on the formation of Nanofibers from pectin and polyethylene oxide blends. J. Appl. Polym. Sci..

[52] Mirtič J., Balažic H., Zupančič Š., Kristl J. (2019). Effect of Solution Composition Variables on Electrospun Alginate Nanofibers: Response Surface Analysis. Polymers.

[53] Rošic R., Pelipenko J., Kocbek P., Baumgartner S., Bešter-Rogač M., Kristl J. (2012). The role of rheology of polymer solutions in predicting nanofiber formation by electrospinning. Eur. Polym. J..

[54] Rinaudo M., Pavlov G., Desbrières J. (1999). Influence of acetic acid concentration on the solubilization of chitosan. Polymer.

[55] Klossner R.R., Queen H.A., Coughlin A.J., Krause W.E. (2008). Correlation of Chitosan’s Rheological Properties and Its Ability to Electrospin. Biomacromolecules.

[56] El-Naggar M.E., Abdelgawad A.M., Salas C., Rojas O.J. (2016). Curdlan in fibers as carriers of tetracycline hydrochloride: Controlled release and antibacterial activity. Carbohydr. Polym..

[57] Ni Annaidh A., Bruyere K., Destrade M., Gilchrist M.D., Ottenio M. (2012). Characterization of the anisotropic mechanical properties of excised human skin. J. Mech. Behav. Biomed. Mater..

[58] Junker J.P., Kamel R.A., Caterson E.J., Eriksson E. (2013). Clinical Impact Upon Wound Healing and Inflammation in Moist, Wet, and Dry Environments. Adv. Wound Care.

[59] Ousey K., Cutting K., Rogers A.A., Rippon M.G. (2016). The Importance of Hydration in Wound Healing: Reinvigorating the clinical perspective. J. Wound Care.

[60] Oryan A., Kamali A., Moshiri A., Baharvand H., Daemi H. (2018). Chemical crosslinking of biopolymeric scaffolds: Current knowledge and future directions of crosslinked engineered bone scaffolds. Int. J. Biol. Macromol..

[61] Stie M.B., Gatke J.R., Wan F., Chronakis I.S., Jacobsen J., Nielsen H.M. (2020). Swelling of mucoadhesive electrospun chitosan/polyethylene oxide nanofibers facilitates adhesion to the sublingual mucosa. Carbohydr. Polym..

[62] Khan T.A., Peh K.K., Ch’ng H.S. (2000). Mechanical, Bioadhesive Strength and Biological Evaluations of Chitosan films for Wound Dressing. J. Pharm. Sci..

[63] Iacob A.-T., Drăgan M., Ionescu O.-M., Profire L., Ficai A., Andronescu E., Confederat L.G., Lupașcu D. (2020). An Overview of Biopolymeric Electrospun Nanofibers Based on Polysaccharides for Wound Healing Management. Pharmaceutics.

[64] Altun E., Yuca E., Ekren N., Kalaskar D.M., Ficai D., Dolete G., Ficai A., Gunduz O. (2021). Kinetic Release Studies of Antibiotic Patches for Local Transdermal Delivery. Pharmaceutics.

[65] Graça M.F.P., de Melo-Diogo D., Correia I.J., Moreira A.F. (2021). Electrospun Asymmetric Membranes as Promising Wound Dressings: A Review. Pharmaceutics.

[66] Zhou H., Shi Z., Wan X., Fang H., Yu D.G., Chen X., Liu P. (2019). The Relationships between Process Parameters and Polymeric Nanofibers Fabricated Using a Modified Coaxial Electrospinning. Nanomaterials.

[67] Piipponen M., Li D., Landen N.X. (2020). The Immune Functions of Keratinocytes in Skin Wound Healing. Int. J. Mol. Sci..

[68] Krzyszczyk P., Schloss R., Palmer A., Berthiaume F. (2018). The Role of Macrophages in Acute and Chronic Wound Healing and Interventions to Promote Pro-wound Healing Phenotypes. Front. Physiol..

[69] ISO 10993-5:2009 (2009). Biological Evaluation of Medical Devices-Part 5: Tests for In Vitro Cytotoxicity.

[70] Wang Y., Beekman J., Hew J., Jackson S., Issler-Fisher A.C., Parungao R., Lajevardi S.S., Li Z., Maitz P.K.M. (2018). Burn injury: Challenges and advances in burn wound healing, infection, pain and scarring. Adv. Drug Deliv. Rev..

[71] Abid S., Hussain T., Nazir A., Zahir A., Ramakrishna S., Hameed M., Khenoussi N. (2019). Enhanced antibacterial activity of PEO-chitosan nanofibers with potential application in burn infection management. Int. J. Biol. Macromol..

[72] Matica M.A., Aachmann F.L., Tondervik A., Sletta H., Ostafe V. (2019). Chitosan as a Wound Dressing Starting Material: Antimicrobial Properties and Mode of Action. Int. J. Mol. Sci..

[73] Foster L.J.R., Butt J. (2011). Chitosan films are NOT antimicrobial. Biotechnol. Lett..

[74] Koh T.J., DiPietro L.A. (2011). Inflammation and wound healing: The role of the macrophage. Expert Rev. Mol. Med..

[75] Du B., Lin C., Bian Z., Xu B. (2015). An insight into anti-inflammatory effects of fungal beta-glucans. Trends Food Sci. Technol..

[76] Chang S.H., Lin Y.Y., Wu G.J., Huang C.H., Tsai G.J. (2019). Effect of chitosan molecular weight on anti-inflammatory activity in the RAW 264.7 macrophage model. Int. J. Biol. Macromol..

[77] Rawlingson A. (2003). Nitric oxide, inflammation and acute burn injury. Burns.

