# Supplementary material for: Multifunctional Nanofibrous Dressing with Antimicrobial and Anti-Inflammatory Properties Prepared by Needle-Free Electrospinning
Source: Pharmaceutics. 2021 Sep 21;13(9):1527. doi: 10.3390/pharmaceutics13091527 (PMC8464763; doi:10.3390/pharmaceutics13091527)
Supplement: Supplementary file 1 [file pharmaceutics-13-01527-s001.zip › pharmaceutics-1365769-supplementary.pdf]

pharmaceutics

Supplementary Materials: Multifunctional Nanofibrous Dressing with Antimicrobial and Anti-inflammatory Properties prepared by Needle-free Electrospinning

Laura Victoria Schulte-Werning, Anjanah Murugaia, Bhupender Singh, Mona Johannessen, Rolf Einar Engstad, Nataša Škalko-Basnet, Ann Mari Holsæter

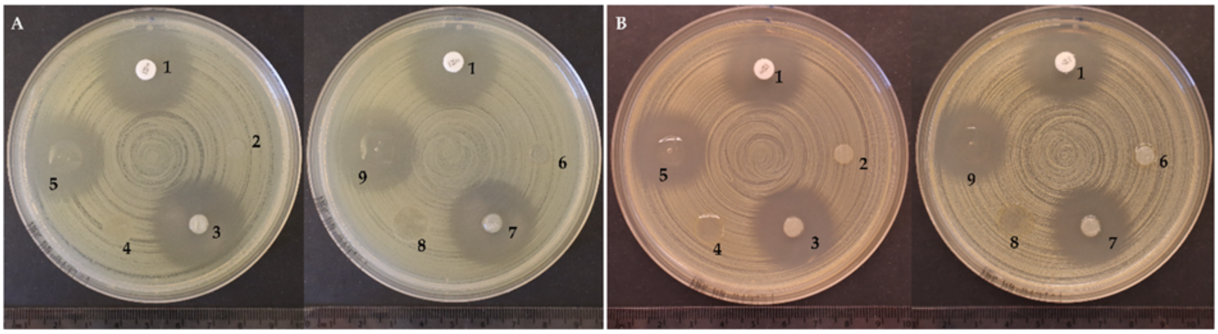

Figure S1. Antibacterial activity of nanofibers. Inhibition of (**A**) E. coli and (**B**) S. aureus by chloramphenicol (1, positive control), (2) βG-CHI-nf, (3) βG-CHI-CAM-nf, (4) βG, (5) βG-CAM-nf, (6) CHI-nf, (7) CHI-CAM-nf, (8) Copol-nf, (9) Copol-CAM-nf. Abbreviations: βG (β-glucan), CHI (chitosan), Copol (co-polymers: polyethylene oxide and hydroxypropylmethylcellulose), CAM (chloramphenicol), nf (nanofiber).

Table S1. Weight of the nanofibrous mats (square, 2 x 2 cm) used for the swelling index testing (n=3).

	**Weight nanofibrous mats (mg)**	
**Batch**	**βG-CHI-CAM-nf**	**CHI-CAM-nf**
**1**	6.3	11.5
	8.9	17.2
	7.2	8.8
**2**	4.5	9.1
	6.7	13.1
	8.8	10.1
**3**	4.3	4.8
	6.5	7.9
	6.7	10.5

Abbreviations: βG (β-glucan), CHI (chitosan), Copol (co-polymers: polyethylene oxide and hydroxypropylmethylcellulose), CAM (chloramphenicol), nf (nanofiber).

Table S2. Weight of the nanofibrous mats (round, diameter = 9 mm) used for the drug release testing (n=2).

	**Weight nanofibrous mats (mg)**			
**Batch**	**βG-CHI-CAM-nf**	**βG-CAM-nf**	**CHI-CAM-nf**	**Copol-CAM-nf**
**1**	9.0	9.6	9.8	8.3
	8.3	7.9	9.2	7.2
**2**	8.3	8.3	9.0	8.9
	8.8	7.9	9.6	8.3
**3**	10.6	9.4	11.5	10.1
	10.2	9.6	10.7	9.9

Abbreviations: βG (β-glucan), CHI (chitosan), Copol (co-polymers: polyethylene oxide and hydroxypropylmethylcellulose), CAM (chloramphenicol), nf (nanofiber).
